# Clinical Application of Circulating Tumor Cells and Circulating Endothelial Cells in Predicting Bladder Cancer Prognosis and Neoadjuvant Chemosensitivity

**DOI:** 10.3389/fonc.2021.802188

**Published:** 2022-02-03

**Authors:** Xiao Yang, Jiancheng Lv, Zijian Zhou, Dexiang Feng, Rui Zhou, Baorui Yuan, Qikai Wu, Hao Yu, Jie Han, Qiang Cao, Min Gu, Pengchao Li, Haiwei Yang, Qiang Lu

**Affiliations:** ^1^ Department of Urology, The First Affiliated Hospital of Nanjing Medical University, Nanjing, China; ^2^ Department of Pediatric Urology, Guangzhou Women and Children’s Medical Center, Guangzhou Medical University, Guangzhou, China

**Keywords:** circulating tumor cells, circulating endothelial cells, bladder cancer, prognosis, neoadjuvant chemosensitivity

## Abstract

**Purpose:**

To investigate the role of circulating rare cells (CRCs), namely, circulating tumor cells (CTCs) and circulating endothelial cells (CECs), in aiding early intervention, treatment decision, and prognostication in bladder cancer.

**Methods:**

A total of 196 patients with pathologically confirmed bladder cancer, namely, 141 non-muscle invasive bladder cancer (NMIBC) and 55 muscle invasive bladder cancer (MIBC) patients. There were 32 patients who received cisplatin-based neoadjuvant chemotherapy (NAC) followed by radical cystectomy (RC). Subtraction enrichment combined with immunostaining-fluorescence *in situ* hybridization (SE-iFISH) strategy was used for CTC/CEC detection. Kaplan–Meier analysis and Cox regression were used to evaluate the overall survival (OS) and recurrence-free survival (RFS). Receiver operator characteristic analysis was used to discriminate NAC sensitivity.

**Results:**

CTCs and CECs were related to clinicopathological characteristics. Triploid CTCs, tetraploid CTCs, and total CECs were found to be higher in incipient patients than in relapse patients (*P* = 0.036, *P* = 0.019, and *P* = 0.025, respectively). The number of total CECs and large cell CECs was also associated with advanced tumor stage (*P* = 0.028 and *P* = 0.033) and grade (*P* = 0.028 and *P* = 0.041). Remarkably, tumor-biomarker-positive CTCs were associated with worse OS and RFS (*P* = 0.026 and *P* = 0.038) in NMIBC patients underwent TURBT. CECs cluster was an independent predictor of recurrence in non-high-risk NMIBC patients underwent TURBT (HR = 9.21, *P* = 0.040). For NAC analysis, pre-NAC tetraploid CTCs and small cell CTCs demonstrated the capability in discriminating NAC-sensitive from insensitive patients. Additionally, tetraploid CTCs and single CTCs elevated post-NAC would indicate chemoresistance.

**Conclusion:**

CTCs and CECs may putatively guide in diagnosis, prognosis prediction, and therapeutic decision-making for bladder cancer.

## Introduction

Bladder cancer is a heterogeneous disease associated with various clinical outcomes. Non-muscle invasive bladder cancer (NMIBC) accounts for roughly 70–80% of bladder cancer and requires routine cystoscopy or even repeated resection (1). Muscle invasive bladder cancer (MIBC) patients have poor prognosis with approximately 50% of patients ultimately suffering from the disseminated micro-metastasis (2). To prevent early dissemination, medically fit patients with clinically localized MIBC are suggested to receive cisplatin-based combination neoadjuvant chemotherapy (NAC). Nonetheless, it still lacks solid biomarkers that can be used to determine whether NAC is necessary or beneficial.

Tissue biopsy is one of the most widely used diagnostic methods for determining the molecular phenotypes of tumors. However, invasive surgical intervention might result in trauma, metastatic risk, and high financial and time cost (3, 4). Liquid biopsy, defined as the capture of tumor-related biomarkers in a liquid sample, has been extensively explored because of its minimal intrusion, low consumption, and convenience of application. When compared to tissue biopsy, the liquid biopsy had the advantage of being easier to repeat over time in order to dynamically monitor disease progression (5). While liquid biopsies have shown potential in identifying MIBC patients for NAC, prospective trials investigating their true clinical applicability for therapy decision making are urgently needed (6, 7).

Circulating tumor cells (CTCs) and circulating endothelial cells (CECs) are the most representative of liquid biopsy due to their minimally invasively detection of cells in carcinoma patients (8). CTCs are malignant epithelial cells derived from primary tumors, representing micro-metastatic disease from the primary tumor or the propensity of evolving disease dissemination (9). CTCs have shown promise for predicting recurrence in high-risk NMIBC (10), evaluating prognosis of RC, and guiding decision-making in bladder-cancer adjuvant chemotherapy (11). However, the relevance of CTCs in NAC decision making remains unclear. CECs originate from the endothelial-cell detachment of the vessel wall and reflect endothelial injury. They have also been proposed as surrogate biomarkers for malignant cancers including colorectal, breast, pancreatic and lung cancers (12). Apart from CTCs/CECs, circulating free DNA (cfDNA) and exosomes are also significant targets for liquid biopsy (13). In bladder cancer patients, cfDNA and exosomes have been implicated in indicating cancer progression or even predicting the drug sensitivity (14, 15).

The most widely used techniques for detecting CTC/CECs include reverse transcriptase-polymerase chain reaction (RT-PCR), immunocytochemistry, flow cytometry immunofluorescence, cytomorphological criteria, and second-generation sequencing (16). Compared with subtraction enrichment combined with immunostaining-fluorescence *in situ* hybridization (SE-iFISH), these traditional detection techniques have different defects like RNA degradation or contamination during RT-PCR, reduced detection sensitivity in flow cytometry and high cost of second-generation sequencing. SE-iFISH integrates all three elements of nucleic acids, proteins, and cell morphology along the cellular bio-chain, allowing for the *in situ* phenotypic identification of tumor biomarkers (TBMs), cell-size identification and karyotypic characterization of chromosomal ploidy in CTCs/CECs (17). According to SE-iFISH analysis, CTCs/CECs are classified into diverse subtypes by chromosome ploidy and their identified TBMs including EpCAM and vimentin (8).

What remains unknown is the relationship between the CTCs/CECs and clinical diagnosis and also pathoanatomical responses to NAC in bladder cancer. The purpose of this study is to explore the utility of CTCs/CECs in the diagnosis and treatment of bladder cancer.

## Materials and Methods

### Study Population

Between November 2016 and October 2019, we enrolled a total of 196 patients at the First Affiliated Hospital of Nanjing Medical University. Patients have to fulfill the following criteria for inclusion: (1) pathologically confirmed bladder cancer, and (2) aged over 18. The major exclusion criteria were as follows: (1) developed other malignancies including upper tract urothelial carcinoma, and (2) refused to sign informed consent. CTC detection was performed on all patients, while CEC detection was performed simultaneously on 133 patients. Peripheral blood (6 ml) was collected prior to any treatment. Written informed consent forms were signed by all patients. The study was approved by the affiliated hospital of Nanjing Medical University (Ethical approval number: 2017-SRFA-016) and performed according to the Declaration of Helsinki principles. The pathological diagnosis was performed by the Union for International Cancer Control TNM classification system (2009), and grade was determined according to the World Health Organization (WHO) 2004 grading of urothelial papilloma (18). High-risk tumors were defined as including any of the following: ① T1 tumor, ② high-grade tumor, ③ CIS, and ④ multiple, recurrent, and large (>3 cm) TaG1G2/LG tumors (all features must be present) (19).

### CTC and CEC Enrichment, Identification and Classification

SE-iFISH approach was utilized to enrich and identify CTCs and CECs. SE-iFISH is a novel approach for detection CTCs/CECs that combines differential phase enrichment, tumor-labeled immunofluorescence staining and i-FISH techniques. It utilizes differential phase enrichment to separate and enrich CTCs/CECs. The enriched CTCs/CECs were then subjected to tumor-labeled immunofluorescence staining and chromosomal fluorescence *in situ* hybridization simultaneously.

**Figure 1:**
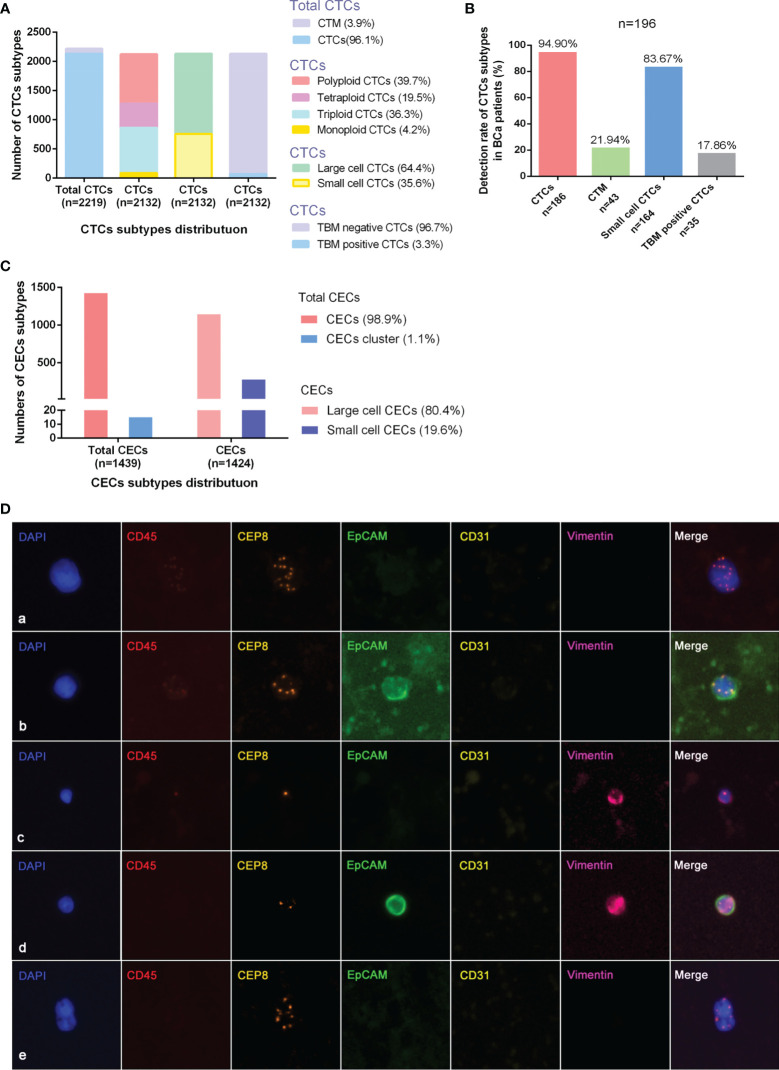
Detection^+^ and characteristics of CTCs and CECs by SE-iFISH in bladder-cancer patients. **(A)** Quantitative composition of diverse CTC subtypes among the total number of CTCs. **(B)** Detective rate distribution of CTC subtypes among all bladder-cancer patients. **(C)** Quantitative composition of CEC subtypes among the total number of CECs. **(D)** Identification of CTCs by SE-iFISH. Line a: Polyploid, large cell, and TBM negative CTCs; Line b: Polyploid, large cell, and TBM-positive (EpCAM**^+^**) CTCs; Line c: Monoploid, small cell, and TBM-positive (vimentin**^+^**) CTCs; Line d: Diploid, small cell, and TBM-positive (EpCAM**^+^**/vimentin^+^) CTCs; Line e: CTM.

CTCs/CECs were enumerated as non-hematopoietic that had not CD45 expressed on the cell surface (CD45^−^) while aneuploid CECs showed positive CD31 expression on the cell surface (CD31^+^) and aneuploid CTCs did not express CD31 (CD31^−^) but TBMs. The identification criteria of CTCs were as follows (**Figure 1D**): aneuploid chromosome 8 with nucleus DAPI^+^/CD45^−^/CD31_−_ or diploid chromosome 8 with nucleus DAPI^+^/CD45_−_/CD31_−_ but positively immunofluorescent with two tumor TBMs (EpCAM^+^/vimentin^+^ and EpCAM^+^/vimentin^−^). Diploid chromosome 8 cells with EpCAM^−^/vimentin^+^ were not defined as CTCs due to vimentin being expressed often in white blood cells. The identification criteria of CECs included the following: aneuploid chromosome 8 with nucleus DAPI^+^/CD45^−^/CD31^+^. Two or more single CTCs or CECs grouped to produce a circulating tumor microembolus (CTM) or CEC cluster. CTC can be divided into large-cell and small-cell CTCs depending on their size in comparison to white blood cells and the same for the CECs. CTCs were classified as monoploid CTC, triploid CTC, tetraploid CTC, and polyploid CTC (≥5 ploidy CTC) according to the karyotypes of chromosomes 8. Depending on whether TBMs can be detected, CTCs can be divided into tumor-biomarker (TBM)-positive CTCs and TBM-negative CTCs. TBM-positive CTCs are EpCAM positive and/or vimentin positive CTCs. CTC positive was defined as CTC number ≥3 and/or CTM ≥1, whereas CEC positive was defined as CECs number ≥2 and/or CEC cluster ≥1.

### Neoadjuvant Chemotherapy Regimen and Assessment

Generally, 70 mg/m^2^ cisplatin-based NAC regimens consisting of cisplatin combined with 1.0 g/m^2^ gemcitabine protocols were administered. NAC was recommended for patients with extravesical disease (≥cT2N0M0). At least two cycles of NAC were performed before RC. Blood was collected from patients prior to or after two cycles of NAC. To evaluate the clinical response, Response Evaluation Criteria in Solid Tumors guideline (Version 1.1) was used. Magnetic resonance imaging was conducted to determine the diameter and volume of tumors prior to and after NAC. Two radiologists were involved to assess tumor response using MRI. T2-weighted imaging, diffusion weighted imaging (DWI), and apparent diffusion coefficient (ADC) were implemented to evaluate therapeutic response to NAC.

### Follow-Up Regimen

Outpatient service and phone calls were used for follow-up. For RC, patients were usually seen every 3 months during the first year following surgery, and every 6 months from the second to fifth years. Follow-up included history, serum, and urine chemistry evaluation, and also physical examination. Every 6 months, abdomen imaging including the urinary tract (CT or MRI of the abdomen/pelvis with intravenous contrast) and chest radiography were conducted. For TURBT, patients received an additional cystoscopy and urinary cytology every 3 months in the first year following surgery and every 6 months from the second to fifth years. The endpoints of the study were as follows: (1) overall survival (OS), defined as the time period between operation and death from any cause, and (2) recurrence-free survival (RFS), defined as the time period between operation and local failure or distant metastases.

### Statistical Analysis

IBM SPSS package (SPSS Inc., Chicago, IL, United States) and GraphPad Prism software (GraphPad Prism Software Inc., San Diego, CA, United States) were used for all statistical analyses. Correlations between the CTC/CEC-positive rate and clinicopathological variables were analyzed by chi-square test. For continuous variables, Student’s t-test was used to compare normally distributed variables or the Wilcoxon rank-sum test was used to compare not normally distributed variables. The area under the curve (AUC) of different CTC subtypes in discriminating chemosensitivity was determined using receiver operator characteristic (ROC) analysis. The change in CTCs between pre- and post-NAC was analyzed by the paired-sample t-test. Kaplan–Meier survival plots were generated based on the numbers of different subtypes of CTCs/CECs, and survival curves were compared using log-rank tests. Hazard ratios (HRs) were derived from univariate and multivariate Cox proportional-hazard regression models. *P <*0.05 was statistically significant, and all statistical analyses were two-sided.

## Results

### Analysis of Quantified CTC and CEC Subtypes

The quantitative distribution of CTC subtypes according to different classification criteria among the entire CTCs is depicted in **Figure 1A**. The detected rate of CTC subtypes distribution indicated that CTCs, small cell CTCs, CTM, and TBM-positive CTCs were present in 94.90, 83.67, 21.94, and 17.86% of all 196 patients, respectively (**Figure 1B**). The distribution of CECs subtypes is depicted in **Figure 1C**.

### CTCs and CECs in Relation to Clinicopathological Characteristics

**Figure 2:**
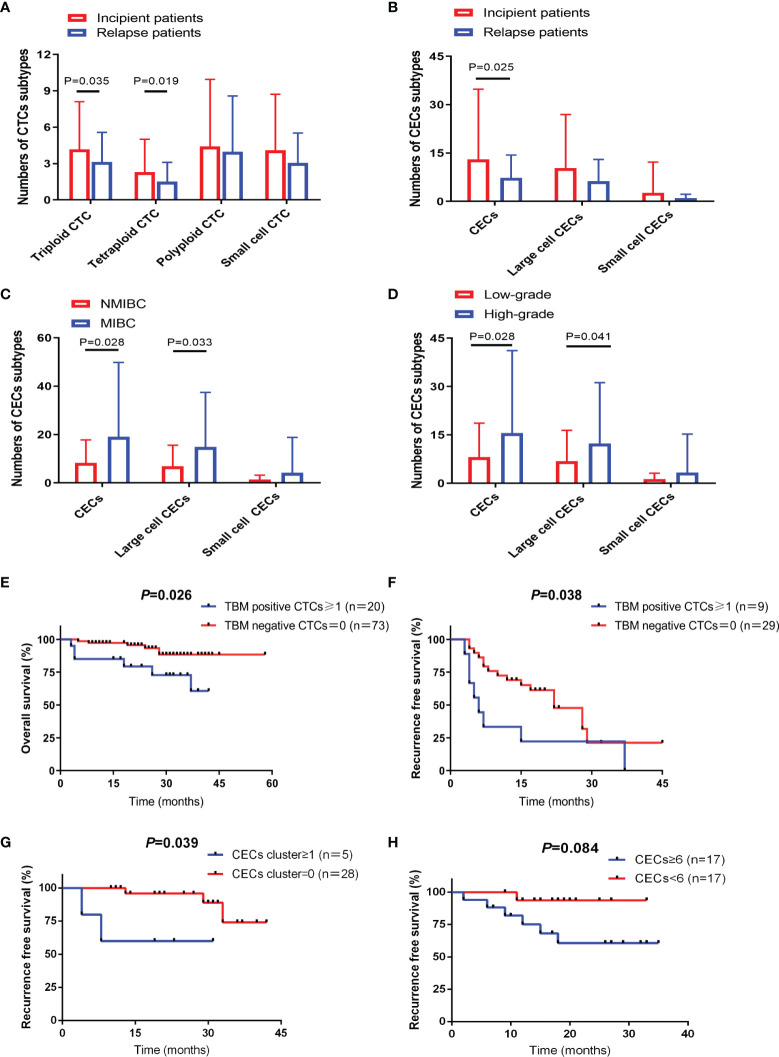
CTC and CEC subtypes correlated with different clinical characteristics and prognosis. **(A)** Distribution of CTC subtypes in incipient and relapse bladder-cancer patients. **(B)** Distribution of CEC subtypes in incipient and relapse bladder-cancer patients. **(C)** Distribution of CEC subtypes in bladder-cancer patients according to tumor stage. **(D)** Distribution of CEC subtypes in bladder-cancer patients according to tumor grade. **(E)** TBM-positive CTC number ≥1 showed poor prognosis with shorter OS in NMIBC patients receiving TURBT. **(F)** TBM-positive CTC number ≥1 showed poor prognosis with shorter RFS in high-risk NMIBC patients receiving TURBT. **(G)** CEC cluster number ≥1 showed poor prognosis with shorter RFS in high-risk NMIBC patients receiving TURBT. **(H)** CECs number ≥6 showed poor prognosis with shorter RFS in MIBC patients receiving RC.

Positive CTCs were present in 163 patients (83.2%), while positive CECs were detected in 105 patients (78.9%). No significant association was found between CTC/CEC positive rates and clinicopathological variables (**Table 1**). However, subgroup analyses revealed that incipient patients exhibited a higher level of triploid and tetraploid CTCs than relapse patients (*P* = 0.036 and *P* = 0.019) (**Figure 2A**).

**Table 1:** Relationship between CTC/CEC positive rate and clinicopathological variables.

Variables	CTCs						CECs					
	Total	Positive		Negative		*P*	Total	Positive		Negative		*P*
	n	n	%	n	%		n	n	%	n	%	
Total	196	163	83.2	33	16.8		133	105	78.9	28	21.1	
Gender												
Male	159	131	82.4	28	17.6	0.549	113	92	81.4	21	18.6	0.097
Female	37	32	86.5	5	13.5		20	13	65.0	7	35.0	
Age												
≥66	107	91	85.0	16	15.0	0.440	73	57	78.1	16	21.0	0.787
<66	89	72	80.9	17	19.1		60	48	80.0	12	20.0	
Tobacco smoking												
Yes	85	70	82.4	15	17.6	0.791	66	55	83.3	11	16.7	0.218
No	111	93	83.8	18	16.2		67	50	74.6	17	25.4	
Alcohol drinking												
Yes	67	59	88.1	8	11.9	0.187	49	43	87.8	6	12.2	0.057
No	129	104	80.6	25	19.4		84	62	73.8	22	26.3	
Stage												
NMIBC	141	117	83.0	24	17.0	0.912	90	72	80.0	18	20.0	0.667
MIBC	55	46	83.6	9	16.4		43	33	76.7	10	23.3	
Grade												
PUNLMP	8	8	100	0	0	0.376	3	3	100	0	0	0.459
Low	95	77	81.1	18	18.9		65	49	75.4	16	24.6	
High	93	78	83.9	15	16.1		65	53	81.5	12	18.5	
Lymph node metastasis												
Yes	8	8	100	0	0	0.194	6	4	66.7	2	33.3	0.450
No	188	155	82.4	33	17.6		127	101	79.5	26	20.5	
Bladder cancer history												
Incipient	43	37	86.0	6	14.0	0.567	29	22	75.9	7	24.1	0.645
Relapse	153	126	82.4	27	17.6		104	83	79.8	21	20.2	

Incipient patients also had an increased CECs level compared to relapsing patients (*P* = 0.025) (**Figure 2B**). In addition, the number of total CECs and large-cell CECs were also associated with higher tumor stage (*P* = 0.028 and *P* = 0.033) (**Figure 2C**) and grade (*P* = 0.028 and *P* = 0.041) (**Figure 2D**).

### CTCs and CECs Contributed to Predicting Oncological Outcomes

Among the 196 patients, 22 were lost to follow-up, 55 patients (31.6%) experienced recurrence and 24 patients (13.8%) died. CTCs demonstrated no significant association between NMIBC and MIBC patients, however, the number of CECs elevated significantly in MIBC patients (**Supplementary Table 1**).

#### TBM-Positive CTCs Predicted Poor Prognosis in NMIBC Patients Receiving TURBT

TBM-positive CTCs served as an unfavorable predictor of OS in NMIBC patients treated with TURBT (**Figure 2E**). Subsequently, we respectively evaluated the prognostic significance in high-risk NMIBC patients and non-high-risk NMIBC patients (**Supplementary Table 2**). As for high-risk NMIBC patients undergoing TURBT, RFS was significantly reduced in the TBM-positive CTC group (**Figure 2F**). While for non-high-risk NMIBC patients undergoing TURBT, there was no significant association between CTCs and prognosis (**Supplementary Table 3**).

#### CEC Cluster Showed a Reduced RFS in NMIBC Patients Receiving TURBT

Univariate analysis of CECs for OS and RFS prediction in all NMIBC patients receiving TURBT failed to reach statistical significance (**Supplementary Table 4**). Whereas, in non-high-risk NMIBC patients receiving TURBT, survival analysis demonstrated that positive CEC cluster did shorten RFS further (**Figure 2G**). While for MIBC patients treated with RC, CECs number ≥6 also showed a shorter RFS but without statistical difference (**Figure 2H**).

#### Association Between CTCs and NAC Response Rate

**Figure 3:**
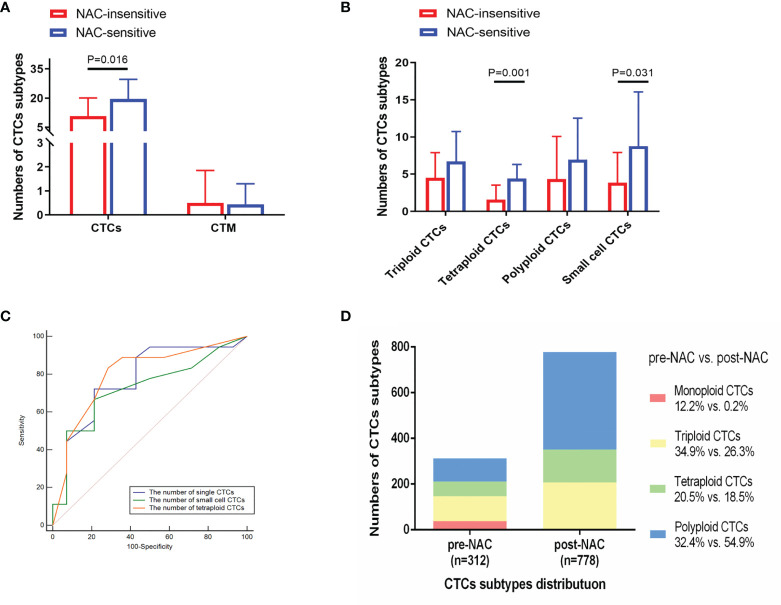
Analysis of CTC subtypes in correlation with NAC sensitivity in 32 bladder-cancer patients. **(A, B)** NAC-sensitive patients had an increasing quantity of pre-NAC CTCs, tetraploid CTCs, and small cell CTCs compared with NAC-insensitive patients. **(C)** Receiver operator characteristic (ROC) curves analysis showed that pre-NAC CTCs, tetraploid CTCs, and small cell CTCs could differentiate NAC-sensitive patients (n = 18) from NAC-insensitive ones (n = 14). **(D)** Distribution of CTC subtypes before and after NAC.

CTCs were quantified in a total of 32 patients prior to NAC followed by RC. A total of 18 of 32 patients were considered responsive, while the remaining 14 were considered insensitive. Pre-NAC single CTCs (P = 0.016), tetraploid CTCs (P = 0.001), and small cell CTCs (P = 0.031) were positively correlated with sensitivity to NAC (**Figures 3A, B**). ROC analysis was also performed to evaluate the ability of tetraploid CTCs, small cell CTCs, and CTCs in discriminating NAC-sensitive patients from resistant patients (**Figure 3C**). Results showed that the potential AUCs were 0.80 for tetraploid CTCs (95%CI = 0.62–0.92, *P <*0.001), 0.72 for small cell CTCs (95%CI = 0.54–0.87, *P* = 0.015), and 0.77 for CTCs (95%CI = 0.59–0.90, *P* = 0.002).

Afterwards, a total of 22 patients received CTC detection after two cycles of NAC. A total of 14 of 22 were considered responsive, and the remaining 8 were considered insensitive. The proportion of polyploid CTCs elevated with the other three karyotypes CTC numbers declined (**Figure 3D**). Compared to 45 patients receiving RC without NAC, NAC group was associated with a decreased number of triploid, tetraploid and small cell CTCs (P = 0.032, P = 0.004, and P = 0.022) (**Supplementary Table 5**). Then we evaluated the consistency between CTC dynamics pre- and post-NAC with the NAC response rate, but no significant association was observed in the 22 patients (**Supplementary Table 6**). Increased count of tetraploid CTCs and single CTCs following NAC indicated its ineffectiveness (P = 0.042 and P = 0.031) (**Table 2**).

**Table 2:** CTC subtypes and dynamics in bladder-cancer patients with neoadjuvant chemotherapy.

CTCs Subtypes	Sensitive	Insensitive	*P*
	(n = 14)	(n = 8)	
Single	−0.29 ± 15.80	58.75 ± 75.89	**0.031**
Monoploid	−2.29 ± 6.62	−0.50 ± 1.41	0.853
Triploid	0.79 ± 7.20	10.63 ± 16.54	0.159
Tetraploid	0.00 ± 4.39	10.00 ± 15.75	**0.042**
Polyploid	1.21 ± 7.48	38.63 ± 51.29	0.074
Small cell	0.21 ± 8.45	11.00 ± 15.95	0.065
TBM positive	0.36 ± 1.45	2.75 ± 7.40	0.432
CTM	−0.21 ± 0.70	1.75 ± 4.65	0.179

Bold values provided in Table 2 means they are significant p values which were less than 0.05.

## Discussion

The detection of CTCs/CECs is the most representative of liquid biopsy in carcinoma patients (20). The clinical implications of CTCs have been reported in bladder cancer; however, most of them are based on conventional strategies and provide conflicting results (21). CellSearch^®^, which utilized an immunomagnetic technique to detect EpCAM^+^ CTCs, was the only FDA-approved CTC-detection platform (22), but it was always associated with a low CTC detection rate (23, 24). Additionally to this method, size-based filtration technique could considerably improve the detection efficiency (25). While all these EpCAM based techniques inevitably underestimate the quantity of CTCs due to missing EpCAM^−^ CTCs (26). Therefore, additional molecule markers like vimentin were investigated and exploited during CTCs detection (26, 27). In present study, SE-iFISH is anticipated to facilitate elucidating how these distinct categories CTCs/CECs functionally interplay with tumor angiogenesis and therapy (8).

With regard to clinical characters analysis, we found that incipient bladder-cancer patients exhibited more triploid CTCs, tetraploid CTCs and total CECs than relapsing patients. One possible explanation is that relapsing patients might receive more routine review than incipient ones, allowing physicians to detect tumors earlier. Of note, our data highlighted the positive correlation between CECs and advanced tumor stage and grade. These data indicated that CECs were more easily detected in more advanced bladder cancer.

As reported, the epithelial to mesenchymal transition (EMT) has been proven to play a role in the tumorigenic process (28). EMT may facilitate the cancer cells to disseminate from local tumors penetrate blood vessels to become CTCs (29–31). CTCs could be classified as epithelial, mesenchymal, or epithelial–mesenchymal hybrids (32). Epithelial markers (EpCAM and E-cadherin) and mesenchymal markers (vimentin and Twist) were used frequently in CTCs detection (33, 34). In the present study, TBM-positive CTCs were defined in the study as EpCAM positive and/or vimentin-positive CTCs. Consistent with a previous study, our results also indicated a relatively low detection rate of TBM-positive CTCs (<20%) (35). To our knowledge, the present study had the largest sample size focusing exclusively on NMIBC rather than T1HG or high-risk NMIBC only. We discovered for the first time that TBM-positive CTCs might be used to predict the prognosis of NMIBC patients. The NMIBC patients receiving TURBT who harbored detectable TBM-positive CTCs were at significantly increased risk of overall mortality. Gazzaniga et al. stated similar results that the presence of CTCs is associated with a short time to the first recurrence in 44 NMIBC patients (36). Notably, NMIBC is heterogeneous cancer and most CTC-related studies on NMIBC have concentrated on T1HG or high-risk NMIBC patients (10, 37). Consistently, according to our analysis on high-risk NMIBC patients receiving TURBT, TBM-positive CTC number ≥1 showed poor prognosis with shorter RFS. Other new biomarkers like PD-L1 expression in CTCs are also in hopes of expanding the role of liquid biopsy in cancer patients (38). Interestingly, BCa patients with type 2 diabetes mellitus (T2DM) suffered a higher risk of recurrence (39). T_2_DM and hyperglycemia have been shown to facilitate EMT process in various cancers (40–42). Therefore, we speculate that hyperglycemia may interfere with the production and detection of CTCs.

However, results data on the actual predictive and prognostic value of CECs in bladder cancer are scarce. Cox regression analysis in our study indicated that positive CEC cluster was an independent risk factor for non-high-risk NMIBC patients treated with TURBT. Intriguingly, although not statistically significant, CEC subtypes predicted poor prognosis of patients receiving RC. Hence, CEC subtypes would also be key players in bladder-cancer diagnosis and prognosis. And our report is also the first research to explore the utility of CECs in bladder cancer.

The cisplatin-based NAC is recommended, while it could provide a minor OS benefit of 5–6% after 10 years (43). Besides, non-responder patients with MIBC have a poor overall survival and are delayed in receiving effective treatment (44). Soave et al. found that bladder cancer patients with the presence of CTCs are more likely to receive adjuvant chemotherapy (11). Our results showed that tetraploid CTCs were the most predictive of NAC sensitivity, followed by single CTCs and small cell CTCs. We also investigated the predictive value of CTC dynamic variation before and after NAC treatment, and found that NAC-insensitive patients possessed an evident elevation of CTCs post-NAC. The number of polyploid CTCs increased most remarkably post-NAC. Thus, we postulated that polyploid CTCs could be an important factor in NAC insensitivity as it was more difficult to eliminate than other CTC subtypes. Interestingly, the most CTC subtypes were elevated post-NAC, which might be attributed to NAC induced shedding of tumor cells into the circulatory system. Several focal tumor cells will necrotize during the initial chemotherapy cycles, which could result in decreased tumor cell adhesion. Thus, the residual tumor cells might be discharged into the bloodstream. This phenomenon was also observed in breast cancer studies (45, 46). Besides, in metastatic urothelial carcinoma, Fina et al. also found that unfavorable trend of CTCs number alterations during chemotherapy may be useful to predict worse prognosis (47). From the above, CTCs may enable the evaluation of NAC response and may hold promise in screening of NAC sensitive patients, thereby allowing NAC insensitive patients to undergo RC earlier. To our knowledge, this study is the first to explore the association between CTCs and bladder cancer NAC.

The Vesical Imaging-Reporting and Data System (VI-RADS) has been demonstrated to accurately predict muscle invasion of BCa before operation (48–51). Although no correlation between CTCs/CECs and BCa stage was observed, the combination of VI-RADS and CTCs/CECs detecting may offer us a more valid judgment basis prior to surgery, particularly for patients with a VI-RADS score of 3. Apart from BCa, other prognostic factors also played a significant role in other hematuria-related genitourinary diseases, such as prostate cancer (52) and prostate surgery associated bleeding (53).

It should be noted that the results of relevant Cox analyses may be insufficiently significant due to the limited number of patients included, especially those who received NAC. While SE-iFISH is a novel CTC detection technique, its application is still limited compared to more established detection methods. As a result, the detection stability and consistency need to be further verified.

## Conclusion

Taken together, based on SE-iFISH strategy, the amounts of single CTCs, small cell CTCs, and tetraploid CTCs could predict NAC sensitivity. Furthermore, the present study established for the first time a relationship between CECs and their subtypes with pathological stage, grade, and clinical outcome. Overall, our findings revealed that various CTCs/CECs subtypes may have diverse potential to guide the diagnosis, prognosis prediction, and therapeutic decisions in bladder cancer, but further analytical validations are still required.

## Data Availability Statement

The original contributions presented in the study are included in the article/**Supplementary Material**. Further inquiries can be directed to the corresponding authors.

## Ethics Statement

All procedures conduct in studies involving human participants comply with the ethical standards of institutions and/or national research committee, as well as with the 1964 Helsinki Declaration and its subsequent amendments or comparable ethical standards.

## Author Contributions

Conception: XY, PL, HYa, and QL. Interpretation or analysis of data: DF, RZ, BY, QW, QC, HYu, JH, and JCL. Preparation of the manuscript: XYu, DF, JL, and ZZ. Revision for important intellectual content: XY, DF, QL, HYa, and PCL. Supervision: QL, HYa, XY, PL, and MG. All authors contributed to the article and approved the submitted version.

## Funding

This work was supported by the National Natural Science Foundation of China (grant nos. 82072832, 81772711, and 82073306), the “333” project of Jiangsu Province (LGY2016002 and 2018055), the Jiangsu Province’s Key Provincial Talents Program (ZDRCA2016006), the Provincial Initiative Program for Excellency Disciplines of Jiangsu Province (grant No. BE2016791), the Priority Academic Program Development of Jiangsu Higher Education Institutions (grant No. JX10231801) and the Clinical Research Cultivation Program, Construction Program of Jiangsu Provincial Clinical Research Centre Support System (BL2014084).

## Conflict of Interest

The authors declare that the research was conducted in the absence of any commercial or financial relationships that could be construed as a potential conflict of interest.

## Publisher’s Note

All claims expressed in this article are solely those of the authors and do not necessarily represent those of their affiliated organizations, or those of the publisher, the editors and the reviewers. Any product that may be evaluated in this article, or claim that may be made by its manufacturer, is not guaranteed or endorsed by the publisher.
