# Supplementary material for: Clinical Application of Circulating Tumor Cells and Circulating Endothelial Cells in Predicting Bladder Cancer Prognosis and Neoadjuvant Chemosensitivity
Source: Front Oncol. 2022 Feb 3;11:802188. doi: 10.3389/fonc.2021.802188 (PMC8851236; doi:10.3389/fonc.2021.802188)
Supplement: Supplementary file 1 [file Table_1.docx]

**Supplementary Table 1 Relationships between the numbers of CTC and CEC parameters and NMIBC/MIBC**

| **CTCs subtypes** | **NMIBC**  **(n=141)** | **MIBC**  **(n=55)** | ***P*** |
| --- | --- | --- | --- |
| Single | 10.80±9.98 | 11.10±9.32 | 0.850 |
| Monoploid | 0.45±2.35 | 0.45±1.41 | 0.998 |
| Triploid | 3.84±3.70 | 4.24±3.61 | 0.494 |
| Tetraploid | 2.21±2.73 | 1.91±1.95 | 0.463 |
| Polyploid | 4.25±5.40 | 4.49±5.24 | 0.773 |
| Small cell | 3.77±4.21 | 4.09±4.44 | 0.641 |
| Large cell | 7.03±7.74 | 7.00±6.83 | 0.981 |
| TBM positive | 0.49±1.48 | 0.33±0.88 | 0.220 |
| CTM | 0.39±1.19 | 0.18±0.61 | 0.448 |
| **CECs subtypes** | **NMIBC**  **(n=90)** | **MIBC**  **(n=43)** | ***P*** |
| CECs | 8.27±9.50 | 19.14±30.69 | **0.028** |
| Large cell | 6.88±8.71 | 14.93±22.49 | **0.028** |
| Small cell | 1.39±1.78 | 4.19±14.64 | 0.219 |
| CEC cluster | 0.12±0.52 | 0.09±0.37 | 0.740 |

**Supplementary Table 2.** **Relationships between the numbers of CTC and CEC parameters and high-risk/non-high-risk NMIBC**

| **CTCs subtypes** | **non-high-risk NMIBC**  **（n=80）** | **high-risk NMIBC**  **（n=61）** | ***P*** |
| --- | --- | --- | --- |
| Single | 11.26±9.21 | 10.20±10.95 | 0.532 |
| Monoploid | 0.39±1.60 | 0.54±3.09 | 0.703 |
| Triploid | 4.16±3.79 | 3.41±3.56 | 0.232 |
| Tetraploid | 2.23±2.63 | 2.18±2.88 | 0.924 |
| Polyploid | 4.40±5.01 | 4.05±5.91 | 0.704 |
| Small cell | 3.61±3.51 | 3.98±5.00 | 0.606 |
| TBM positive | 0.38±1.25 | 0.41±1.13 | 0.863 |
| CTM | 0.66±1.88 | 0.26±0.60 | 0.077 |
| **CECs subtypes** | **non-high-risk NMIBC**  **(n=39)** | **high-risk NMIBC (n=51)** | ***P*** |
| CECs | 9.31±10.14 | 7.74±9.00 | 0.366 |
| Large cell | 7.49±8.95 | 6.41±8.57 | 0.564 |
| Small cell | 1.82±2.15 | 1.06±1.36 | 0.058 |
| CEC cluster | 0.21±0.69 | 0.06±0.31 | 0.227 |

**Supplementary Table 3. Univariate analysis for CTCs predicting OS and RFS in non-high-risk NMIBC patients receiving TURBT (n=55)**

| **CTCs subtypes** | **OS** | | | **RFS** | | |
| --- | --- | --- | --- | --- | --- | --- |
|  | **HR** | **95%Cl** | ***P*** | **HR** | **95%Cl** | ***P*** |
| Single (≥9 vs. <9) | 52.148 | —— | 0.341 | 55.447 | 0.511-6013 | 0.093 |
| Triploid (≥4 vs. <4) | 2.879 | 0.299-27.706 | 0.360 | 2.196 | 0.659-7.313 | 0.200 |
| Tetraploid (≥2 vs. <2) | 2.181 | 0.227-20.977 | 0.500 | 1.409 | 0.423-4.689 | 0.576 |
| Polyploid (≥3 vs. <3) | 43.626 | —— | 0.377 | 3.336 | 0.729-15.278 | 0.121 |
| Small cell (≥3 vs. <3) | 0.759 | 0.107-5.390 | 0.782 | 1.121 | 0.354-3.551 | 0.846 |
| CTM (≥1 vs. 0) | 2.708 | 0.379-19.372 | 0.321 | 0.735 | 0.197-2.734 | 0.646 |
| TBM positive (≥1 vs. 0) | 2.636 | 0.512-25.830 | 0.197 | 1.063 | 0.287-3.936 | 0.927 |

**Supplementary Table 4. Univariate analysis for CECs predicting OS and RFS in NMIBC patients receiving TURBT (n=61)**

| CECs subtypes | OS | | | RFS | | |
| --- | --- | --- | --- | --- | --- | --- |
|  | HR | 95%Cl | *P* | HR | 95%Cl | *P* |
| CECs (≥6 vs. <6) | 0.764 | 0.108-5.435 | 0.788 | 0.521 | 0.219-1.214 | 0.141 |
| Large cell (≥5 vs. <5) | 1.109 | 0.156-7.874 | 0.918 | 0.475 | 0.191-1.182 | 0.110 |
| Small cell (≥2 vs. <2) | 0.479 | 0.050-4.612 | 0.524 | 0.705 | 0.284-1.749 | 0.451 |
| CECs cluster (≥1 vs. 0) | 0.042 | —— | 0.658 | 1.981 | 0.579-6.780 | 0.276 |

**Supplementary Table 5. CTC subtypes and bladder patients treated with RC with and without NAC (n=67)**

| **CTCs subtypes** | **Without NAC**  **(n=45)** | **With NAC**  **(n=22)** | ***P*** |
| --- | --- | --- | --- |
| Single | 10.12±9.69 | 21.24±29.33 | 0.104 |
| Monoploid | 0.51±2.06 | 1.81±5.46 | 0.303 |
| Triploid | 3.53±2.82 | 5.29±3.45 | **0.032** |
| Tetraploid | 1.47±1.89 | 3.52±2.68 | **0.004** |
| Polyploid | 4.60±6.07 | 10.62±27.15 | 0.327 |
| Small cell | 2.98±2.93 | 7.24±7.71 | **0.022** |
| TBM positive | 0.31±1.22 | 0.10±0.30 | 0.429 |
| CTM | 0.24±0.61 | 1.10±2.61 | 0.154 |

**Supplementary Table 6. CTC subtypes and dynamics in bladder-cancer patients with neoadjuvant chemotherapy**

| **CTCs subtypes** | **Pre-NAC**  **(n=22)** | **Post-NAC**  **(n=22)** | ***P*** |
| --- | --- | --- | --- |
| Single | 14.18±10.75 | 35.36±52.87 | 0.821 |
| Monoploid | 1.73±5.35 | 0.09±0.29 | 0.104 |
| Triploid | 4.95±3.61 | 9.32±12.32 | 0.312 |
| Tetraploid | 2.91±2.37 | 6.55±18.88 | 0.415 |
| Polyploid | 4.59±5.06 | 19.41±34.87 | 0.157 |
| Small cell | 5.82±6.45 | 9.68±12.33 | 0.601 |
| TBM positive | 0.18±0.40 | 1.50±4.51 | 0.101 |
| CTM | 0.59±1.26 | 1.09±2.49 | 0.572 |
